# Knowledge and practices of cardiopulmonary arrest and anaphylactic reactions in the radiology department

**DOI:** 10.4102/sajr.v24i1.1841

**Published:** 2020-05-28

**Authors:** Sarah K. Osiemo, Callen K. Onyambu, Angeline A. Aywak

**Affiliations:** 1 Department of Radiology, The Mater Hospital, Nairobi, Kenya; 2 Department of Diagnostic Imaging and Radiation Medicine, Faculty of Medicine, University of Nairobi, Nairobi, Kenya

**Keywords:** cardiopulmonary arrest, anaphylactic reactions, management, radiologists, residents, radiographers

## Abstract

**Background:**

Emergencies in the radiology department may arise in critically ill patients who are brought to the department for imaging, interventional procedures or as a result of adverse reactions to contrast media used for imaging. Adverse reactions to contrast media range from minor to severe life-threatening effects and initial, prompt management decreases complications. Radiology staff must possess knowledge of the management of anaphylactic or anaphylactoid contrast reactions and cardiopulmonary arrest (CPA) as they are likely to be the first responders.

**Objectives:**

To determine the knowledge and practices amongst radiologists, radiology residents and radiographers in the management of CPA and adverse reactions to contrast media.

**Method:**

This cross-sectional study was performed between March and August 2016 at Kenyatta National Hospital using a questionnaire.

**Results:**

Eighty participants were enrolled. None answered all the questions correctly, with only 55% of radiologists, 35% of residents and 39% of radiographers scoring above 50%. The majority (82%) of participants had adequate knowledge regarding the symptoms, signs and risk factors of adverse reactions to contrast media; however, only 30% knew that intravenous epinephrine is the recommended therapy for a severe anaphylactic reaction. Shortcomings in terms of adequate training were found in this study, with the majority of respondents having not attended any life support course in the preceding 5 years.

**Conclusion:**

Health providers within the radiology unit had knowledge about identifying both mild and severe symptoms of anaphylactic reactions. However, there were knowledge gaps regarding the management of these reactions.

## Introduction

Cardiopulmonary arrest (CPA) refers to the cessation of cardiac activity and breathing, leading to hemodynamic collapse and interruption of pulmonary gaseous exchange.^1^ Resuscitation is a pivotal lifesaving skill, which, if administered quickly and correctly, greatly improves the chances of survival following CPA.

The practice of cardiopulmonary resuscitation (CPR) has evolved over the years with significant changes that have a great impact on the patient’s outcome. The American Heart Association’s (AHA) basic life support (BLS) and advanced cardiac life support (ACLS) algorithms are widely accepted as standard resuscitation guidelines. Basic life support forms the foundation for the management of CPA and emphasises high-quality CPR and use of an automated external defibrillator.^2^

Advanced cardiac life support focuses on additional algorithms tailored to deal with different causes of cardiac arrest, like ventricular fibrillation, ventricular tachycardia, pulseless electrical activity and asystole. Emphasis is also given to the use of drugs, for example, epinephrine, advanced airway support and recognition and treatment of reversible causes of CPA.

Iodine-based contrast media and gadolinium chelates are the main contrast agents used to enhance the differentiation of tissues during imaging. Adverse effects, although infrequent, can occur after contrast administration. The prevalence of such effects has been shown to be 0.6% for iodinated contrast media^3,4^ and 0.12% for gadolinium-based contrast agents.^5^ These adverse effects can be classified into organ specific and non-organ specific, acute and delayed reactions. The acute reactions can be divided into anaphylactoid or idiosyncratic and physiologic or non-idiosyncratic reactions. Further subdivision into mild, moderate and severe is based on severity, with mild reactions being more common (71%).^4^ There are recognised risk factors that increase the likelihood of the occurence of adverse reactions, for example, prior allergic-like reaction to intravenous contrast media, asthma and significant cardiovascular disease.^6^ However, the majority are non-specific.

Despite their infrequent occurrence, the radiology department staff members need to be aware of the presentation, risk factors and management of these conditions to ensure optimal patient care. The American College of Radiology (ACR) has set up guidelines that govern the management of such reactions depending on their severity. The main aim of this study was to assess the theoretical knowledge and practice amongst the radiology staff, based on the most current BLS, ACLS and ACR guidelines.

## Methods

In this observational, cross-sectional study, a total of 82 participants were selected; however, two declined to contribute. The participants included radiologists, radiology residents and radiographers in the radiology department of a tertiary teaching and referral hospital. The study was carried out from 01 March to 31 August 2016. A sample size of 82 was calculated using the formula for estimating prevalence in cross-sectional studies (Cochran 1963) with a precision of 0.05, and assuming a prevalence of 50%, a 95% level of confidence and a finite population of 103 radiology healthcare providers.

A simple random sampling method was used to select the participants, excluding visiting staff and those who declined consent. Data collection was performed through administration of a validated quantitative questionnaire, which was completed in the presence of the researcher. There were five questions allotted to demographic data. There were also 15 multiple-choice and six closed-ended questions assessing the knowledge and practice of CPR, as well as the recognition and management of contrast media reactions. These were based on the current BLS, ACLS and ACR guidelines updated in 2015, 2016 and 2015, respectively.

Data collected were analysed using the Statistical Package for the Social Sciences (SPSS) version 20.0 for Windows® and Chi-squared tests were performed to determine the statistical significance (*p* < 0.05) of the results.

### Ethical consideration

The study was approved by the Kenyatta National Hospital/University of Nairobi Ethics and Scientific Review Committee (Reference no.: KNH-ERC/A/51 and approval number P763/12/2015). All the participants signed an informed consent form before participating in the study.

## Results

The total number of participants was 80, of which 18 (22.5%) were consultant radiologists, 39 (48.8%) were residents and 23 (28.8%) were radiographers. The ages of the participants ranged between 23–65 years. There were 43 (53.8%) male and 37 (46.3%) female participants. None of the participants answered all the questions correctly, with only 55% of radiologists, 35% of residents and 39% of radiographers scoring above 50%.

Among the 18 consultants interviewed, seven had more than 15 years of working experience, seven had between 1 and 4 years and the rest had between 5 and 15 years of experience. The majority (43.5%) of the radiographers had between 1 and 4 years of working experience. The residents, being in a 4-year training programme, had between 1 and 4 years of experience in the radiology department, with 12 of the 38 being in their second year of training.

The majority of the participants (83%) had attended at least one life support training course but not in the preceding 5 years. There was a significant association between health workers’ level of expertise and their attendance of life support courses (*p* = 0.024) (Table 1).

**TABLE 1:** Life support training course attendance according to health providers’ level of expertise.

Health worker cadre	Life support training								*p*
	BLS only		ACLS only		BLS and ACLS		None		
	*n*	%	*n*	%	*n*	%	*n*	%
Consultant	6	33.3	0	0.0	7	38.9	5	27.8	0.024
Radiology resident	8	20.5	3	7.69	22	56.4	6	15.4	-
Radiographer	14	60.9	0	0.0	7	30.4	2	8.7	-

BLS, basic life support; ACLS, advanced cardiac life support.

## Cardiopulmonary resuscitation and contrast media anaphylaxis knowledge

**FIGURE 1:**
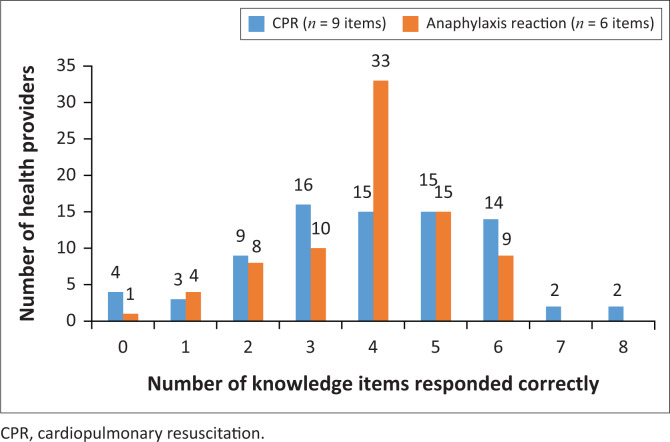
Number of correct health worker responses to knowledge items regarding cardiopulmonary resuscitation and anaphylaxis reaction.

Nine items on CPR and six anaphylaxis reaction items were used to assess participants’ knowledge. The responses to these knowledge questions are presented in Figure 1. In terms of comparison of the two areas, participants displayed higher knowledge scores in anaphylaxis reactions than in CPR. The modal (most frequent) score on anaphylaxis reactions was 4 out of 6, with 33 (41.3%) participants scoring four items correctly. Nine (11.3%) participants scored all six anaphylaxis reaction items correctly. For CPR knowledge, the modal score was 3 out of 9 correct items (*n* = 16 [20%]).

### Knowledge of cardiopulmonary resuscitation

Regarding the knowledge of CPR and the current ACLS and BLS guidelines, at least half of the participants responded correctly to four of the nine items: head tilt-chin lift manoeuvre for opening the airway (63; 78.8%), appropriate to administer rescue breaths if pulse is present (54; 67.5%), appropriate to initiate compressions if pulse is absent (51; 63.8%) and correct adrenaline administration (1 mg) during cardiac arrest (43; 53.8%).

Of the respondents, 77.8% incorrectly provided the BLS sequence as airway-breathing-compression (A-B-C) instead of compression-airway-breathing (C-A-B) and only 36.7% gave the correct chest compression to rescue breaths ratio at 30:2.

Knowledge was not significantly associated with the level of expertise (*p* = 0.364) or surprisingly, the years since attendance of life support training (*p* = 0.128). Knowledge was however significantly associated with the years of work experience (*p* = 0.016), with most of the participants who had 10 or more years of experience scoring at least 50%, compared to those with 1–4 years of experience (38.2%) and 5–9 years of experience (0%) (Table 2).

**TABLE 2:** Cardiopulmonary resuscitation knowledge scores according to participants’ years of practice (*p* = 0.016).

Years of practice	CPR knowledge score			
	> 50%		< 50%	
	*n*	%	*n*	%
1–4 years	21	38.2	34	61.8
5–9 years	0	0.0	7	100.0
10–15 years	4	50.0	4	50.0
> 15 years	7	77.8	2	22.2

Note: Cardiopulmonary resuscitation is a procedure to support and maintain breathing and circulation for a person who has stopped breathing and/or whose heart has stopped.

CPR, cardiopulmonary resuscitation.

### Knowledge of anaphylactic reactions to contrast media

The majority of participants (82%) had adequate knowledge of the symptoms, signs and risk factors of adverse reactions to contrast media; however, only 24 (30%) of the 80 participants correctly gave epinephrine as the recommended drug for the treatment of severe anaphylaxis. The majority of participants (60%) incorrectly gave hydrocortisone as the drug of choice.

For the remaining items, good performance was noted, with 82% of participants correctly responding to the questions: pruritus is a symptom of mild anaphylactic reaction, laryngeal oedema is a symptom of severe anaphylactic reaction and asthma is a risk factor for adverse contrast reactions. There was a significant association between health workers’ level of expertise (*p* < 0.001) and years of practice (*p* < 0.05) with knowledge of anaphylaxis. There was a significant association between the level of expertise (*p* < 0.001) and knowledge of anaphylaxis, with regard to the majority of radiology residents (35; 89.7%) and consultants (15; 83.3%) scoring at least 50% compared to the radiographers (7; 30.4%) who also scored 50%. Knowledge of contrast anaphylaxis was significantly (*p* < 0.05) associated with the years of practice, with all health workers with >15 years’ experience scoring at least 50%.

Attendance of life support training and the year of attendance was not significantly associated with anaphylaxis knowledge (*p* = 0.86 and *p* = 0.251, respectively). Amongst the anaphylactic reaction knowledge items that showed poor health worker understanding, the radiographers scored the least knowledge. Only 21.7% of radiographers knew that epinephrine is the drug of choice compared to 33.3% of residents and radiologists.

### Practices

Of the 80 participants, 32 (40%) reported that they had seen a patient experiencing an adverse reaction to contrast within the radiology department. Seven (20.6%) of the health workers who had managed a patient experiencing an adverse reaction indicated that they had done so according to ACR guidelines.

There were 31 (38.8%) participants who had ever witnessed a patient experiencing a CPA in the radiology department, and in 23 (71.9%) of cases, BLS/ACLS guidelines were applied for patient management. Most of the participants (51; 63.8%) reported that they knew where the emergency trolley was located within the department; however, only 10 (12.5%) checked it before performing radiological procedures.

## Discussion

This research has shown that the theoretical knowledge of BLS and ACLS pertaining to the management of CPA amongst radiologists, residents and radiographers is poor to moderate. More than half of the respondents (58.8; 57.8%) scored <50% in the assessment of CPR knowledge and none answered all questions correctly. This was an unexpected finding as available evidence from high-quality systematic reviews suggests that life support knowledge and skills decay within 6 months to 1 year after training and that skills decay faster than knowledge.^7^ For anaphylaxis knowledge, the performance was good, with 71.3% (57 out of 80) of health workers responding correctly to at least four of the six questions on adverse reactions.

The mean score on the knowledge questions was 52.4%, which was below the recommended pass mark by the AHA. Our finding is similar to that of O’Neill et al.^8^ who reported that their study participants had a mean score of 50%, which was below the acceptable level set at 70%. This was because of inadequate training and lack of refresher courses, a finding similar to the current study in which 50.6% of the staff members had not received any ACLS training.

The attendance of life support courses was not significantly associated with knowledge (*p* > 0.05), nor was the duration since the last training. This is consistent with the study finding by Alam et al.^9^ who found that only 28% of those who had attended a life support course more recently were more likely to respond correctly. In addition, the lack of course attendance on knowledge in the present study could be explained by two factors: rapid decay of knowledge and duration since attendance of these courses.

Recent changes in guideline recommendations impacted the performance in knowledge assessment. For example, in 2000, AHA changed the recommended ratio of chest compressions: ventilation from 15:2 to 30:2.^10^ In this study, only 36% responded to this question correctly, with those who had attended life support training before 2005 more likely to indicate a ratio of 15:2. Tapping et al.^11^ also found that respondents who had done life support training prior to 2005 stated the CPR ratio to be 15:2. Cardiopulmonary resuscitation steps are another area of resuscitation training that was recently revised and also examined in this study. In 2010, the AHA re-arranged the order of CPR steps from A-B-C to C-A-B.^12^ This question was amongst the most poorly answered, with only 15% giving the correct response.

The current findings highlight the need for refresher training because of the constant evolution of life support algorithms with significant changes and the documented rapid decay of knowledge acquired in life support courses. This is of particular concern in this study, as most of the participants who had attended a BLS course had done so more than 5 years before this study. The same applied to ACLS attendance. Schellhammer et al.^13^ found that only 41.8% of the radiologists in their study had ever attended training courses and of these, 69% were trained > 5 years prior to the study.^13^ The AHA recommends that the interval between initial CPR training and a refresher course should not be more than 2 years; however, studies evaluating the deterioration of CPR skills amongst medical personnel have shown a decline at 2–12 months following completion of training.^14,15,16,17,18,19^

With regard to contrast media reactions and management, most of the radiologists, residents and radiographers performed relatively well in the identification of mild and severe anaphylactic reactions and in their knowledge of the risk factors for an adverse reaction. At least 82% of health workers correctly identified a symptom of mild reaction, a symptom of severe reaction and a risk factor for anaphylactic reaction. However, responses on pharmacological management of severe reactions were poor. Health workers commonly stated that they would administer hydrocortisone as the drug of choice (60%), with only 30% correctly identifying epinephrine as the drug of choice and 28.8% stating the correct dose.

This shows that there is a great misconception regarding the drug of choice in the emergency management of severe contrast anaphylaxis. Our findings are in contrast to that of Lightfoot et al.^20^ who had 91% of the radiologists choosing epinephrine as the most important medication for the management of a severe contrast reaction; however, none provided the correct dosage. The disparity in findings could be attributed to the fact that our study included radiographers (28.8%), with the majority achieving diploma and higher diploma qualifications. There is limited emphasis on the pharmacological management of contrast reactions in radiographer training. In our data, this group consistently scored lower than radiologists and residents in identifying the recommended drug of choice for contrast reactions and its dose. Even after excluding radiographers, the clinicians (residents and radiologists) in our setting were approximately 60% less likely to correctly identify the most important medication compared to Canadian and American radiologists, possibly reflecting inadequate recent training in BLS and ACLS.

In general, radiology staff members are not involved in the practice of acute clinical medicine and do not manage emergencies on a day-to-day basis unlike their physician counterparts. This could explain why the questions on knowledge of the recommended drug (epinephrine) and its correct dose were poorly answered.

The need for prompt and effective CPR and management of CPA cannot be over-emphasised as it is known to significantly decrease morbidity and mortality.^21^ Of the patients brought to the radiology department, some may deteriorate and suffer CPA because of their primary condition or as a result of a severe anaphylactic reaction to contrast media.^22^ In this study, the majority of respondents (71.9%) who had witnessed CPA within the department managed the patient according to BLS/ACLS guidelines. This is similar to the study conducted by Schellhammer et al.,^13^ who found that most of the radiologists had performed at least one CPR during their time of practice. Similarly, few (38.8%) of the respondents had witnessed a patient experiencing an adverse reaction to contrast media and most did not provide management according to the guidelines provide by the ACR.

The low rate of observation of these reactions is attributed to the liberal use of non-ionic contrast media which have a lower risk of complications. Also, most of the respondents were radiology residents who had been in the radiology department for less than 4 years and thus were more likely to have witnessed fewer, if any, reactions. The low response on the use of the recommended ACR guidelines is probably because of the infrequent and inadequate training on these reactions and their management.

Most (63.8%) of the participants knew where the emergency trolley was located in the department and these findings are similar to that of Lightfoot et al.,^20^ where 62% participants knew the location of the emergency drug epinephrine in the computed tomography imaging rooms. Despite awareness of the location of the emergency trolley, very few of the respondents (12.5%) checked it before performing any procedure in the department. This translates to a lack of knowledge regarding the contents of the trolley and may create a crisis when an emergency arises. Lightfoot et al.^20^ also found that only 11% knew the concentration of the epinephrine in their emergency trolleys and the type of equipment stocked for its administration.

## Conclusion

Healthcare providers within the radiology unit had knowledge of identifying both mild and severe symptoms of anaphylactic reactions to contrast media. There were however knowledge gaps regarding the management of such reactions. They also demonstrated inadequate knowledge of the fundamental and critical components of BLS and ACLS.

The fact that the knowledge of lifesaving skills is inadequate amongst the health workers in the radiology department is alarming and there is a need for encouraging formal training (BLS and ACLS) and awareness of evidence-based recommendations contained in clinical guidelines. This should be supplemented with regular in-service training and re-certification to ensure retention of knowledge.
